# No neighbour-induced increase in root growth of soybean and sunflower in mesh-divider experiments after controlling for nutrient concentration and soil volume

**DOI:** 10.1093/aobpla/plab020

**Published:** 2021-04-14

**Authors:** Bin J W Chen, Li Huang, Heinjo J During, Xinyu Wang, Jiahe Wei, Niels P R Anten

**Affiliations:** 1 College of Biology and the Environment, Nanjing Forestry University, Longpan Road 159, Xuanwu District, Nanjing 210037, China; 2 Section of Ecology and Biodiversity, Institute of Environmental Biology, Utrecht University, Padualaan 8, 3584CH Utrecht, The Netherlands; 3 Centre for Crop Systems Analysis, Wageningen University, P.O. Box 430, 6700AK Wageningen, The Netherlands

**Keywords:** Game theory, ideal free distribution, mesh divider, neighbour detection, root competition, tragedy of the commons

## Abstract

Root competition is a key factor determining plant performance, community structure and ecosystem productivity. To adequately estimate the extent of root proliferation of plants in response to neighbours independently of nutrient availability, one should use a set-up that can simultaneously control for both nutrient concentration and soil volume at plant individual level. With a mesh-divider design, which was suggested as a promising solution for this problem, we conducted two intraspecific root competition experiments: one with soybean (*Glycine max*) and the other with sunflower (*Helianthus annuus*). We found no response of root growth or biomass allocation to intraspecific neighbours, i.e. an ‘ideal free distribution’ (IFD) norm, in soybean; and even a reduced growth as a negative response in sunflower. These responses are all inconsistent with the hypothesis that plants should produce more roots even at the expense of reduced fitness in response to neighbours, i.e. root over-proliferation. Our results suggest that neighbour-induced root over-proliferation is not a ubiquitous feature in plants. By integrating the findings with results from other soybean studies, we conclude that for some species this response could be a genotype-dependent response as a result of natural or artificial selection, or a context-dependent response so that plants can switch from root over-proliferation to IFD depending on the environment of competition. We also critically discuss whether the mesh-divider design is an ideal solution for root competition experiments.

## Teaser

Game-theoretical models predict that plants should overinvest in root growth beyond the communal optimum in competition for soil resources. This phenomenon is often coined as a ‘tragedy of the commons’. To test this hypothesis, we conducted two greenhouse experiments on intraspecific root competition using a mesh-divider design to control for both nutrient concentration and soil volume in two common crop species, soybean and sunflower. In contrast to this hypothesis, we showed that in mesh-divided below-ground competition, soybean plants grew roots regardless of neighbours, and sunflower plants even had a tendency to underinvest in root growth.

## Introduction

Root competition for soil resources is a ubiquitous feature in terrestrial plant communities (de Kroon *et al.* 2003). It is also one of the fundamental forces determining the structure and dynamics of both natural and managed ecosystems (Kiær *et al.* 2013; Aschehoug *et al.* 2016). Its impacts on the growth and productivity of plants at both individual and group levels have long been an important topic in plant ecology (Cahill and McNickle 2011) and agriculture (Weiner 2019).

The potential selective advantage of resource investment in root production for a plant engaged in root competition can be analysed using evolutionary game theory (Gersani *et al.* 2001; McNickle and Dybzinski 2013; Cabal *et al.* 2020). Game-theoretical models predict that when resource investment in root production of a plant is based on an assessment of cost and benefit balance in response to the decline of nutrient availability caused by the consumption from both the plant and its neighbours, the plant should over-proliferate roots to an extent that exceeds the optimal level for maximized performance (e.g. seed production) in below-ground competition with neighbours (Gersani *et al.* 2001). Such phenomena have been coined as a ‘tragedy of the commons’ (TOC; Hardin 1968). The first empirical evidence of a TOC root response came from Gersani *et al.* (2001) using soybean (*Glycine max*). To bypass the confounding effects of variation in resource availability on root growth embedded in the traditional competition set-up (i.e. one plant in one pot as solitary treatment vs. two plants in one pot as neighbour treatment, given a fixed amount of nutrients per pot), they adopted a ‘split-root’ design that can provide the same amount of nutrients per plant for both solitary and neighbour treatments by growing one plant in one pot for the former, and two plants sharing two pots for the latter, at the same nutrient concentration. They found that soybean plants interacting with neighbours produced 85 % more root mass but 30 % less seed mass than those grown alone. This finding seemed to support the idea of a TOC in root growth, but subsequent studies have shown mixed results. Some of them were confirmatory also finding a TOC root response to neighbours (Maina *et al.* 2002; O’Brien *et al.* 2005; Zhu *et al.* 2019); while others, for instance, found their results to better fit an ‘ideal free distribution’ (IFD) response, i.e. a norm describing that root growth is simply based on nutrient availability regardless of neighbours (Semchenko *et al.* 2007; Markham and Halwas 2011; McNickle and Brown 2014a). As this deals with a fundamental aspect of our understanding of diversity and coexistence in natural communities (Vincent and Brown 2005) with potentially far-reaching consequences for agriculture (Anten and Vermeulen 2016; Weiner *et al.* 2017), further experiments trying to elucidate the processes underlying these differences are urgently needed.

Meanwhile, there is also an ongoing debate on the interpretation of root over-proliferation in the above-mentioned studies, mainly arising from biases associated with the experimental methods used. A key point raised about split-root designs is that by keeping both nutrient amount and concentration per plant constant, it doubles soil volume for plants in neighbour treatments as compared to those in solitary treatments (Hess and de Kroon 2007; Semchenko *et al.* 2007; Chen *et al.* 2012). The greater root production of plants observed in neighbour treatments could then simply be a result of more space to grow roots rather than a direct response to competition (Hess and de Kroon 2007). Indeed, recent evidence showed that plants use a ‘chemical radar’ (e.g. by sensing the extent of diffusion of root-secreted ethylene) to detect below-ground obstacles (Pandey *et al.* 2021), and regulate the level of root production as well as above-ground growth in response to available soil volume (Wheeldon *et al.* 2021). To control for this so-called volume effect, Chen *et al.* (2015) conducted a split-root experiment across a range of volumes, and surprisingly, observed an under-proliferation of roots in response to neighbours. However, their design in turn could not control for differences in nutrient concentration between neighbour and solitary treatments (McNickle 2020). Therefore, only when nutrient amount, nutrient concentration and soil volume are simultaneously controlled, can one adequately test for the root investment strategy of plants for resource harvest in below-ground competition.

**Figure 1.**
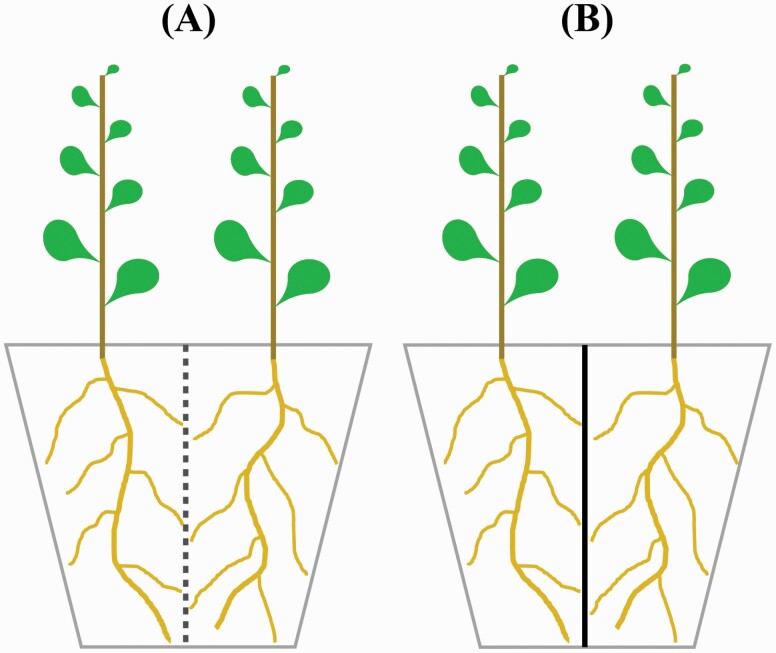
Illustration of the experimental design for (A) neighbour treatment, i.e. two plants in one pot were divided by a food-grade nylon mesh that can prevent the pass of roots but allow the exchanges of liquids and substances between two soil compartments; and (B) solitary treatment, i.e. two plants in one pot were divided by a plastic film that can prevent any form of exchanges between the two compartments.

Notably, a prerequisite for the occurrence of TOC root responses predicted by game theory is the sharing of at least part of a common resource pool between plants (i.e. two plants compete for the common resources; Gersani *et al.* 2001; McNickle and Dybzinski 2013). This implies that even without direct or close root contact (e.g. intermingling of roots), below-ground competition between two plants can still occur as long as nutrients can move (by diffusion) between two soil spaces each occupied by the roots of one of two plants. Interestingly, a ‘mesh-divider’ design may provide a promising solution (Zhu and Zhang 2013; McNickle 2020). In this set-up both solitary and neighbour treatments are composed of two plants separately grown in two divided compartments of one pot, but with a plastic film divider for the former and a mesh divider for the latter (Fig. 1). The mesh divider only prevents roots from passing but allows other substances (e.g. water, nutrients and root exudates) to move from one compartment to the other. It was thus considered that nutrient competition can still occur between mesh-divided plants (Zhu *et al.* 2019, 2020). The film divider, on the other hand, completely isolates two plants below-ground by blocking all exchanges. With this design, plants in the two treatments experience the same level of nutrient amount and concentration as well as soil volume (McNickle 2020), although the design may introduce other experimental issues (Chen *et al.* 2020).

In the current study, we hypothesize that plants interacting with neighbours should over-proliferate roots at the expense of less seed production. To test for this hypothesis, we conducted two greenhouse experiments on intraspecific root competition using the mesh-divider design to control for both nutrient concentration and soil volume in two common crop species, soybean (*G. max*) and sunflower (*Helianthus annuus*). The former species has already been found to show tendencies towards root over-proliferation (Gersani *et al.* 2001), and the latter one belongs to the Asteraceae family which has been seldom tested in this research field.

## Materials and Methods

The experiments were carried out at a plastic-roofed greenhouse facility of Nanjing Forestry University from early August (summer) to late October (autumn). Commercially available seeds of soybean (cultivar ‘white in August’) and sunflower (a dwarf cultivar ‘363’) were first surface-sterilized by soaking them in a bleach solution (10 % sodium hypochlorite) for 5 min, and then washed and sown in moist sand. Three days later, newly germinated plants were selected and transplanted to seedling trays. After another 5 days of growth, seedlings with similar sizes and healthy appearances of each species were further selected and transplanted to circa 9-L plastic pots filled with vermiculite. To study intraspecific root competition between soybean plants and also between sunflower plants, each pot was divided into two compartments, and in each compartment one seedling was planted (i.e. 4.5 L soil volume per plant). So, each pot contained two plants of the same species. The divider was either made of a food-grade nylon mesh with an aperture size of 48 μm to only prevent root penetration (Fig. 1A), or a plastic (polytetrafluoroethylen, PTFE) film to prevent any exchange between two compartments (Fig. 1B). Thus, the paired conspecific plants would not have any form of root interaction with a film divider (i.e. solitary treatment), while indirect interactions via mass flow and the diffusion of soluble substances with a mesh divider were still possible (i.e. neighbour treatment). By doing so, all plant individuals in the experiment should have the same size of soil volume for root growth. In addition, the distance between two paired conspecific plants was kept at 10 cm to standardize the above-ground interaction. For each species, 24 pots with 48 plants in total (i.e. 12 pairs of plants per treatment) were randomly arranged on the bench to control for environmental variation. Prior to planting, the substrates were saturated with tap water. During the experiment, all plants were carefully watered daily, so that the surface of substrates was maintained moist but without liquid drainage from the pot bottom. In addition, each plant was fertilized with 100 mL Hoagland solution (30 % strength) every other day.

As stated in the previous section, it is critical in our experimental set-up that the mesh divider allows soil water together with nutrient ions move (e.g. by diffusion) between two compartments. Otherwise, there would be no difference in growth conditions (i.e. below-ground isolation) between the two treatments. Although there have been successful examples using the mesh-divider design to study nutrient competition (see citations above), we still performed extra tests that specifically examine the occurrence of diffusion processes (respectively, using soil moisture sensor and soil salinity sensor for the movements of water and nutrient ions) passing through mesh dividers in the neighbour treatment **[see****Supporting Information—Appendix 1****]**. We found clear signs of water and nutrient movements passing through meshes **[see****Supporting Information—Appendix 1****]**, based on 24-h monitoring of the diffusion processes, thus confirming the robustness of our design.

For each species, the experiment was terminated and plants were harvested when seeds ripened and most leaves turned completely yellow but their roots were still alive (i.e. 7 weeks of cultivation for soybean, and 9 weeks for sunflower). During the harvest, plant individuals were separated into root, vegetative shoot (leaves and stem) and reproductive organs (i.e. pods for soybean, flower heads for sunflower). All materials were oven-dried at 65 °C for 72 h before weighing. Then, the seeds were separated from the pods/flower heads and weighed again.

The difference in plant biomass of each species between solitary and neighbour treatments was tested using linear mixed models with root interaction (solitary vs. neighbour) as the fixed factor and pot replicate as the random factor. All parameters except for reproductive and seed mass of sunflower were ln-transformed in the analyses. Regarding the possible role of allometric growth in plants, we further examined the responses of plant vegetative shoot and reproductive mass to root interaction treatments using linear mixed models with root mass as the allometric covariate (root interaction, root mass and their interaction term as the fixed factors, and pot replicate as the random factor). In the allometric analyses, all biomass variables (including both dependent and independent ones) were ln-transformed. All the analyses were performed in R v.3.5.3 (R Core Team 2019) using the lme4 package (Bates *et al.* 2015).

## Results

**Figure 2.**
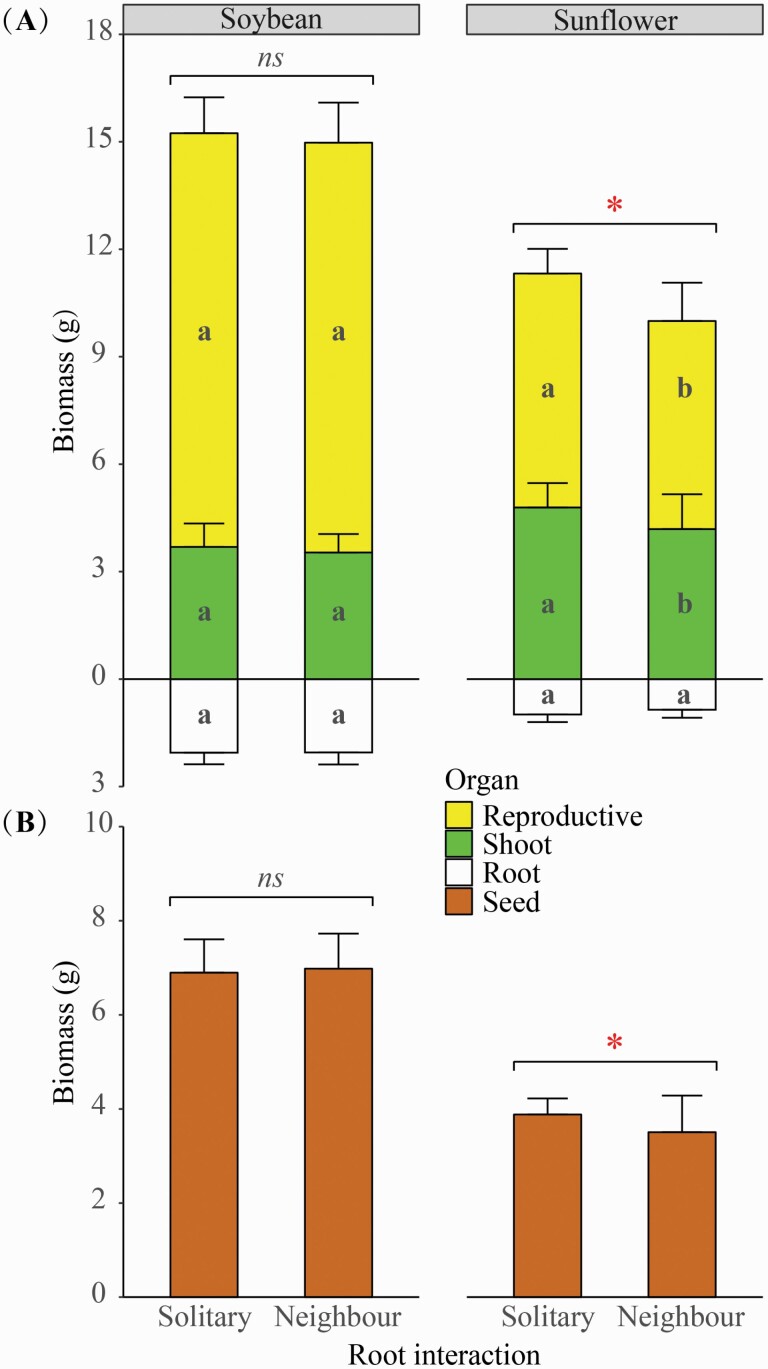
The effects of root interaction (with the use of a plastic film divider as the solitary treatment, and a mesh divider as the neighbour treatment) on the biomass distribution of soybean and sunflower plants. For each species, different letters indicate a significant difference in (A) the mass of reproductive (pod for soybean and flower head for sunflower), shoot or root organs between treatments; */ns denotes a significant/non-significant difference in (A) total or (B) seed mass between treatments. The error bars denote 1 SD of the mean.

For soybean, there was no difference in either plant size (in terms of total mass) or vegetative (i.e. root and shoot mass) or reproductive performance (i.e. pod and seed mass) between the solitary and neighbour treatments (Fig. 2). The same results were obtained when the allometric relationship was considered in the analyses (Table 1).

**Table 1.** Summary of the effects of root interaction (neighbour vs. solitary treatment, or mesh vs. film divider), root mass (as the allometric covariate) and their interaction on plant shoot and reproductive mass in linear mixed models.

	Root interaction (RI)			Root mass (RM)			RI × RM		
	*d.f.*	*F*	*P*	*d.f.*	*F*	*P*	*d.f.*	*F*	*P*
Soybean									
Seed mass	1, 43.99	1.97	0.168	1, 43.63	0.05	0.824	1, 43.63	1.86	0.179
Reproduction^†^	1, 43.90	1.20	0.279	1, 43.99	0.27	0.605	1, 43.99	1.48	0.231
Shoot mass	1, 43.45	0.60	0.442	1, 42.38	6.71	0.013	1, 42.38	0.36	0.553
Sunflower									
Seed mass	1, 44.00	1.88	0.177	1, 44.00	3.08	0.086	1, 44.00	0.31	0.580
Reproduction^‡^	1, 21.99	2.62	0.120	1, 43.98	16.13	<0.001	1, 43.98	0.04	0.846
Shoot mass	1, 22.81	1.07	0.311	1, 43.75	64.38	<0.001	1, 43.75	0.41	0.525

Reproduction for ^†^soybean stands for the mass of pods; while for ^‡^sunflower it is the mass of a whole flower head with all seeds attached. *P*-values are calculated from *F*-statistics using a type III sum of squares, based on the Satterthwaite’s method for the degrees of freedom (*d.f.* here presented as ‘numerator *d.f.*, denominator *d.f.*’). *n* = 48 plants (in 24 pairs) per species.

For sunflower, on the other hand, plants had significantly lower shoot mass (Fig. 2A) and reproductive mass (Fig. 2A and B) in the neighbour treatment than in the solitary treatment. However, this was not a result of root over-proliferation. On the contrary, plant root mass was marginally lower (*P* = 0.053) in the neighbour than in the solitary treatment. The declines of both above- and below-ground growth led to a significant reduction of the total mass of plants in the neighbour treatment (Fig. 2A). However, when the allometric covariate—root mass—was considered in the analyses, differences in plant shoot or reproductive mass between the neighbour and solitary treatments were not significant (Table 1).

## Discussion

Using a mesh-divider design, we controlled for the levels of nutrient amount, nutrient concentration and also soil volume for plants at individual level in both neighbour and solitary treatments. In contrast to our hypothesis, we still found no evidence of root over-proliferation associated with less seed production, i.e. a TOC, in interplant root interaction independently of nutrient availability. Instead, there was no response in soybean, and even a tendency of root under-proliferation of sunflowers growing with neighbours compared to plants growing alone. Below, we discuss possible reasons that may explain deviations in the behaviour of our plants from the response of root over-proliferation.

It is very unlikely that the absence of a TOC response in our soybean plants was due to a lack of below-ground interaction between mesh-divided plants in our neighbour treatment. This is because (i) there is solid evidence that our mesh divider allowed the diffusion of water and nutrients between two compartments **[see****Supporting Information—Appendix 1****]**, so that two mesh-divided plants were still able to compete for soil resources; and (ii) there was a significant reduction in the growth of mesh-divided sunflowers, which resembled a typical sign of allelopathy (see more discussion further down) that can only occur when allelochemicals secreted by neighbours’ roots can pass through mesh dividers in our neighbour treatment. Therefore, this absence of a TOC in our soybean plants may imply that (i) the over-proliferation of soybean roots in the neighbour treatment of Gersani *et al.* (2001) can be at least partially attributable to a larger soil volume (Hess and de Kroon 2007); and (ii) the root foraging behaviour of our soybean cultivar obeyed an IFD response.

Accumulating evidence suggests that TOC responses in interplant root competition may be species-specific (Smyčka and Herben 2017). A number of species, including oat (*Avena sativa*; Semchenko *et al.* 2007), wild pansy (*Viola tricolor*; Lankinen 2008), big bluestem (*Andropogon gerardii*; Markham and Halwas 2011) and field mustard (*Brassica rapa*; McNickle and Brown 2014a), demonstrated an IFD strategy in interplant root competition. More interestingly, some species even showed a shift of rooting behaviour that can vary from TOC to IFD, such as pea (TOC in O’Brien *et al.* 2005; IFD in Mobley *et al.* 2020), common bean (TOC in Maina *et al.* 2002; IFD in Nord *et al.* 2011) and soybean (TOC in Gersani *et al.* 2001; IFD in this study). This intraspecific variation may simply be attributed to the intrinsic difference between varieties (or cultivars) used in different studies, i.e. some studies using varieties that express a TOC response and others using varieties that do not.

Alternatively, it may imply that the occurrence of a TOC response critically depends on the environment of competition arena (McNickle 2020). The differences in experimental set-ups between our study (i.e. mesh-divider design) and that of Gersani *et al.* (i.e. split-root design) may provide some clues. For instance, neighbour treatment in Gersani *et al.* enabled significant intermingling of roots which represented the most thorough and direct way of root competition between plants. With root intermingling, soil resources are fully shared, and any change of nutrient concentration caused by a plant will be detected by the other competing plant in a very short time. Such a situation well fits an important assumption of Gersani *et al.*’s game-theoretically non-spatially-explicit root competition model, in which a unit of resource taken up by a root in any soil location should immediately reduce the resource availability everywhere in the soil space, predicting the TOC responses (Zea-Cabrera *et al.* 2006; O’Brien and Brown 2008; McNickle and Brown 2012; McNickle and Brown 2014b). Our set-up, on the other hand, separated two root systems thus preventing root intermingling. By doing so, the detection of neighbour-caused nutrient decline relies on water/nutrient diffusion in soil between two mesh-divided compartments. Since soil diffusion is a relatively slow process (Nye 1980; also **see****Supporting Information—Appendix 1**) and the efficiency exponentially decreases with distance (Bai *et al.* 2012) especially when two soil compartments have a similar water potential, resources in the two mesh-divided compartments become partially shared, and the time it takes between a neighbour plant taking up nutrients and the focal plant detecting this reduction will be prolonged. Thus, the sensitivity of plants to resource uptake of neighbours will also be inevitably reduced. In other words, the absence of TOC responses in our study could have been due to an incomplete share of resources resulting from a slow rate of soil diffusion between plants in our mesh-divider design.

Moreover, due to intrinsic differences in the mobility of different chemicals (Raynaud and Leadley 2004), mesh-divided plants are more likely to compete for mobile resources, particularly water and nitrate, but not for less mobile ones, such as phosphate (Chen *et al.* 2020). Based on an assumption that root growth is mainly determined by nutrient concentration in local space, a simulation model showed that plants favour a much lower degree of root overlap with neighbours when competing for mobile nitrogen than when competing for immobile phosphorus (de Vries 2013). This would further hinder the occurrence of TOC responses of plants in our mesh-divider design.

A recent study by Cabal *et al.* (2020) showed that when spatial dimension (i.e. a distance-related cost of nutrient transport from soil location to plant stem) is incorporated in Gersani *et al.*’s original model, the new model predicts that plants should overproduce (or underproduce) roots in nutrient patches that are closer to (or further away from) them than to neighbours, due to a relative lower (or higher) cost of nutrient transportation in shorter (or longer) distance than neighbours. This prediction appears to be supported by some empirical observations (Cabal *et al.* 2020; Lepik *et al.* 2021). Their findings indicate that interplant distance (or plant density) is a critical component determining root foraging behaviours of plants in resource competition (Cabal *et al.* 2020), and suggest that an evaluation of root production at whole-plant level or over large spatial scales may lead to incomplete- even miss-understanding of plant–plant root interaction (Semchenko 2020). This also suggests that the differences in results between Gersani *et al.* and us may reflect a difference in spatial pattern of nutrient-transportation cost for plants in resource competition between the two studies (i.e. two closely grown plants had full access to each other’s below-ground territory in the split-root design, but were restricted in their own territories in the mesh-divider design).

However, to what extent aforementioned interpretations hold true needs to be further investigated. For instance, there is evidence that the occurrence of TOC responses at least above-ground does not require resource pool being fully shared. Anten (2002) showed that plants can overinvest in leaf area beyond the communal optimum in competition for light when their canopies are only partially overlapped. Although there is a great number of studies modelling the effects of soil diffusion on the water and nutrient uptake of the plants (Roose *et al.* 2001; Chapman *et al.* 2012), the extent to which diffusion rate and efficiency can determine the outcome of interplant root competition, particularly in a game-theoretical context, is still understudied. Thus, there is a need to further develop game-theoretical root competition models by incorporating soil diffusion effects with spatially explicit analyses (e.g. functional-structure plant models; Evers *et al.* 2019). Despite of these uncertainties, it should be noted that significant TOC root responses were still reported from several studies using mesh-divided wheat (Zhu *et al.* 2019, 2020), and one of them even used meshes with a much smaller aperture size (20 μm; Zhu *et al.* 2020) than ours.

The question arises whether the IFD behaviour of our soybean plants also reflects the fact that nutrient availability provided here (i.e. 100 mL 30 % Hoagland solution [HS] given to 4.5 L soil per plant every other day, which is mathematically equivalent to 33 mL 10 % HS per L soil per plant per day) was too low to manifest a competition response of our plants? It should be noted however that the level of fertilization applied in Gersani *et al.*’s experiment was similarly low (i.e. 400 mL 10 % HS given to 13 L soil per plant every other day, which is mathematically equivalent to 15 mL 10 % HS per L soil per plant per day). We are aware that soybean, as a typical leguminous species, has the potential to gain extra nitrogen from rhizobial symbiosis, which may influence the outcome of nutrient competition. The nodulation condition was not specified in Gersani *et al.*, but the extent of this influence in our study is questionable, as we observed almost no signs of nodulation in our soybean plants during root washing. The might be due to the facts that (i) our seeds were surface-sterilized before sowing; and (ii) the substrate vermiculite is a material that produced by a massive heating process. Though very unlikely, the IFD response of our soybean might also be attributed to a confounding effect of different divider materials used between two treatments.

It was long thought that plants respond to neighbours below-ground only based on a nutrient depletion effect (de Kroon *et al.* 2003). However, evidence in the past two decades accumulates that plants are also able to recognize and distinguish roots between self and a neighbour independently of nutrient availability (so-called ‘neighbour detection’; Chen *et al.* 2012). Root exudates seem to function as the chemical messengers mediating the detection process (Pierik *et al.* 2013). Since the mesh allowed water, nutrients and even allelochemicals diffuse and pass through, it is safe to say that the exchange of root exudates, thus chemical communication including neighbour detection, can occur between mesh-divided soybean plants. Although the incentive for a TOC response predicted by game theory can be simply attributed to a balance between cost and benefit for root investment of a plant in response to the decline of soil nutrient concentration (McNickle and Dybzinski 2013) but not to the presence of a neighbour *per se*, such a prediction has still been adopted as a reasonable hypothesis for testing the effects of neighbour detection (Chen *et al.* 2020). This is because, from an evolutionary perspective the ability of neighbour detection should facilitate plants to accurately compete for soil resources with neighbours rather than with themselves (Chen *et al.* 2012). Unfortunately, the IFD response of our mesh-divided soybean plants was not consistent with the hypothesized root over-proliferation after plant detecting the presence of a neighbour. This may simply imply that our soybean plants were not capable of detecting neighbours.

However, there is a growing body of literature showing that plants are also capable of perceiving the difference in level of relatedness between themselves and their neighbours, and often restrain the expression of their competitive response to relatives in order to maximize their inclusive fitness (also group performance, reviewed by Anten and Chen 2021). This so-called ‘kin recognition’ is believed to more likely evolve in plant populations where genetic variation is small (Platt and Bever 2009). Apparently, the ways of modern crop breeding and management (i.e. massive mono-cropping of single cultivar or even single genotype) fit the criteria well, and indeed kin recognition has been demonstrated in many crop species (Anten and Chen 2021), including soybean (Murphy *et al.* 2017). Thus, there is also a possibility that the IFD response of our soybean plants in response to neighbour treatment reflects a strong effect of kin recognition, which enable them to cooperate rather than compete with their kin (intracultivar) neighbours.

Empirical studies so far have shown that plants can compete for nutrients at least by two means (i) produce more roots to increase its own nutrient uptake, and (ii) inhibit root growth of neighbours via allelopathy to reduce neighbours’ nutrient uptake (Schenk 2006; Zhang *et al.* 2021). The latter strategy has been well documented for sunflowers at both interspecific (Irons and Burnside 1982; Azania *et al.* 2003) and intraspecific levels (i.e. autotoxicity; Singh *et al.* 1999). In addition, regarding the fact that root interaction did not affect the above- and below-ground allometry of sunflowers (Table 1), it seemed that the observed biomass difference between two treatments was mainly caused by the variation in the rate but not the strategy of plant growth. Thus, the growth reduction of our mesh-divided sunflower plants possibly reflected an inhibition effect from autotoxicity caused by root-secreted allelochemicals from conspecific neighbours.

## Conclusion

After controlling for both nutrient concentration and soil volume with a mesh-divider design, we found no evidence for root over-proliferation with less reproduction (i.e. a TOC predicted by game theory), but an IFD response in soybean and even a negative growth response in sunflower as the results of intraspecific root interaction independently of nutrient availability. The soybean results suggest that TOC can be a genotype-dependent, and/or context-dependent response of plants in root competition. The sunflower results imply that an inclusion of more parameters (such as the cost and benefit for chemical interference) in the game-theoretical models may be required to get more insight into plant–plant root interactions. Moreover, the mesh-divider design is probably still not an ideal set-up for testing TOC responses, since the sensitivity of plants to neighbour-induced nutrient depletion can be largely reduced. Interestingly, this ‘disadvantage’ appears to make this design a promising set-up for testing interplant chemical communication in the studies of below-ground neighbour detection, results of which are often confounded by the effect of nutrient competition. Finally, we need to be aware that the mesh-divided root interaction is a highly artificial condition. The extent to which conclusions drawn from the experiments can be directly applied to plant–plant interactions under natural field conditions still needs further investigations.

## Supporting Information

The following additional information is available in the online version of this article—

**Appendix 1.** The tests for the occurrence of solution diffusion in our mesh-divider treatment.

**Data Sheet 1.** The raw data used in the analyses.

plab020_suppl_Supplementary_Appendix_S1

plab020_suppl_Supplementary_Materials

## Data Availability

The raw data are available in **Supporting Information—Data Sheet 1**.

## Notes

**Agroecology & Environment. Chief Editor:** Amélie Gaudin
