# Supplementary material for: No neighbour-induced increase in root growth of soybean and sunflower in mesh-divider experiments after controlling for nutrient concentration and soil volume
Source: AoB Plants. 2021 Apr 14;13(3):plab020. doi: 10.1093/aobpla/plab020 (PMC8112762; doi:10.1093/aobpla/plab020)
Supplement: plab020_suppl_Supplementary_Appendix_S1 [file plab020_suppl_supplementary_appendix_s1.pdf]

Appendix 1. The tests for the occurrence of solution diffusion in our mesh-divider treatment.

The idea of this test is that, since nutrients are given in the form of Hoagland solution (i.e. inorganic chemical ions), if our mesh-divider design does allow the diffusion of nutrients between two compartments, then after watering one compartment (so-called “donor compartment”) with nutrient solution, we should observe an increase of concentration of inorganic chemical ions (which can be measured by a salinity sensor) in the compartment (so-called “receiver compartment”) on the other side of the mesh. Thus, we performed two independent tests that specifically and respectively examined nutrient diffusion between mesh-divided compartments in two conditions, (1) the diffusion of nutrients from a donor compartment to its receiver compartment which is filled with dry vermiculite, so-called “dry test”; and (2) the diffusion of nutrients from a donor compartment to its receiver compartment which is filled with moist vermiculite, so-called “wet test”. We were aware that a remarkable diffusion of water should also occur in the dry test, so we also used a soil moisture sensor to detect the water diffusion process. Thus, the two tests were run as 24-hour monitoring of the changes in soil moisture (for water diffusion) and soil salinity (for nutrient diffusion) in the receiver compartment.

(1) Dry test in the mesh-divider treatment

As illustrated in Figure S1, we re-produced a mesh-divider setup (neighbour treatment) with the same standard used in our formal experiment, but without plants. The donor compartment was filled with vermiculites which were saturated with distilled water in advance, while the receiver compartment was filled with dry vermiculites just < 1 min ahead of the start of monitoring to prevent earlier water diffusion. In the receiver compartment, a soil salinity sensor (range: 0~19.99 mS/cm, resolution: 0.01 mS/cm, error: ± 2%) was buried 5 cm deep from the substrate surface and 2.5 cm away from the mesh; and a soil moisture sensor (range: 0~100%, resolution: 0.1%, error: ≤ 3%) was also inserted into the substrate, with a distance of 2.5 cm from the mesh. The monitoring system (Hangzhou Lvbo Instrument Co., Ltd.) was set to an automatic mode with a time interval of 5 min, which is the minimal interval set for automatic recording. Meanwhile, the system can show real-time data in the screen of the recorder. A real-time data can also be recorded by manually pressing a button on the recorder during the automatic recording. As noted, these sensors are designed for the measurements in real soils, thus their sensitivities and accuracies may be reduced in the vermiculite. The monitoring started at the time point when 100 ml Hoagland solution with a 30%

standard strength was poured on the surface of the donor compartment. In details, the solution was poured along a parallel line that was 2.5 cm away from the mesh (the same way as applied in the formal experiment). The monitoring lasted for 24 hrs.

As shown in Figure S2A, the initial stage of solution diffusion rate in the dry vermiculite was rather low. The first detection of soil moisture at the location of measurement occurred almost one hour after the addition of solution in the donor compartment. However, from then on, the soil moisture increased very fast. After only one more hour, the moisture was already quite close to a stable level. Notably, after 24 hours of water diffusion, all vermiculites in the receiver compartment turned wet (Figure S3). For the diffusion of nutrient ions in the dry test, the first record was 15 min later than the first record of soil moisture. This was probably because only after soil moisture had reached a threshold, the salinity (conductance) could be measured. Interestingly, the detection of salinity disappeared in the later stage of measurements. This might be due to a strong diffusion of water from the saturated donor compartment to the dry receiver compartment. So that the concentration of nutrients was diluted below the threshold for the detection of the salinity sensor.

(2) Wet test in the mesh-divider treatment

The basic setup was the same as described in the dry test. The only difference was that the receiver compartment was also filled with vermiculites that were saturated with distilled water before the test. Results showed that although the moisture of both donor and receiver compartments were at saturation level (may imply limited extent of water diffusion), we still observed significant signs of nutrient diffusion passing through the mesh, as shown in Figure S2B. Only 3 min after the addition of solution in the donor compartment, the salinity sensor had already recorded the first non-zero value. And this value remained to the end of the test.

Therefore, the aforementioned tests showed us clear signs of nutrient diffusion between mesh-divided compartments in both dry and wet conditions, though the detailed diffusion pattern was also influenced by soil moisture condition.

Besides, we also examined whether there was any diffusion (i.e. leakage) between film-divided compartments (i.e. solitary treatment) in dry and wet conditions. Since the monitoring system did not capture any change (i.e. zero values of soil moisture and salinity throughout the dry test; and a zero value of soil salinity throughout the wet test), and we also manually observed no sign of water diffusion (Figure S4). We are confident to say that the answer is no.

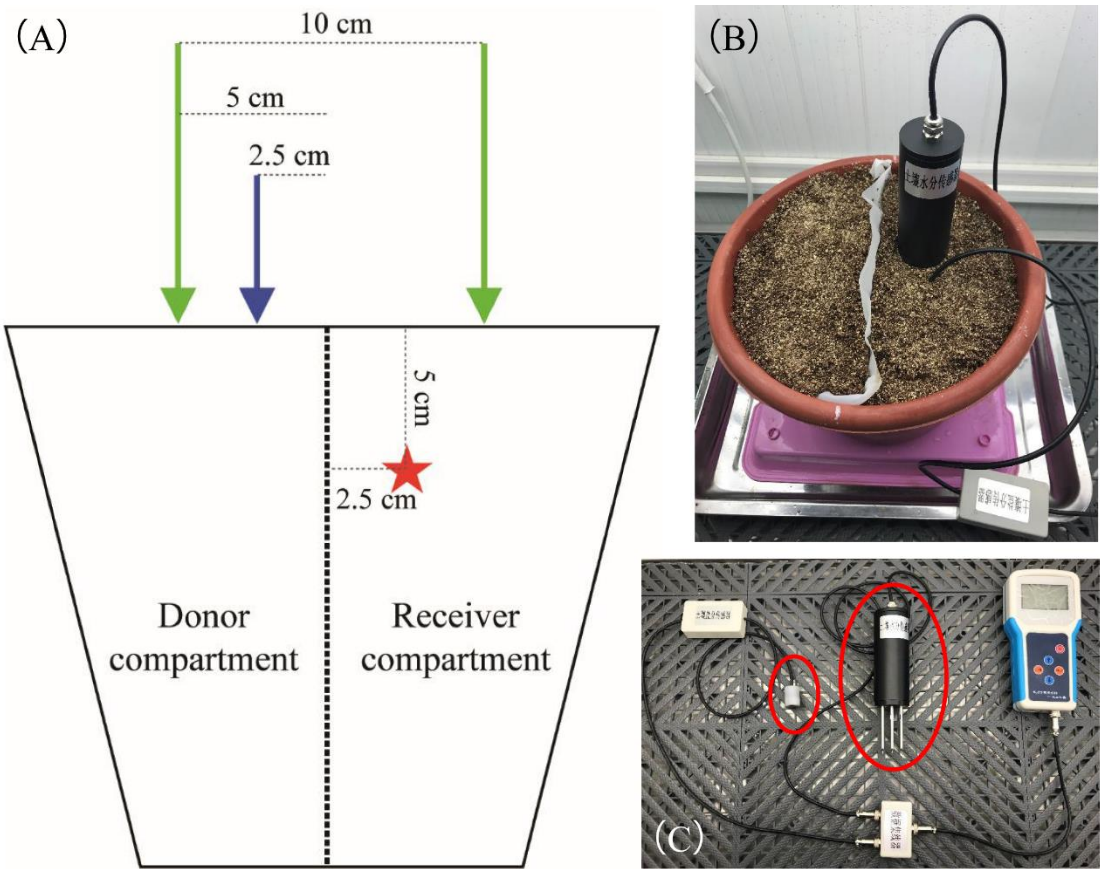

Figure S1. Illustration of the basic setup for examining the occurrence of water and nutrient diffusion between mesh-divided compartments. In (A) the green arrows indicate the locations of plant stems in the formal experiment; the blue arrow indicates the location where 100 ml Hoagland solution (in 30% strength) was poured into the donor compartment; and the red star indicates where the diffusion of nutrients and water was monitored in the receiver compartment. The real scenario of test setup is shown in (B). The monitoring system is shown in (C), with the soil moisture sensor in the large red circle, and the soil salinity sensor in the small red circle.

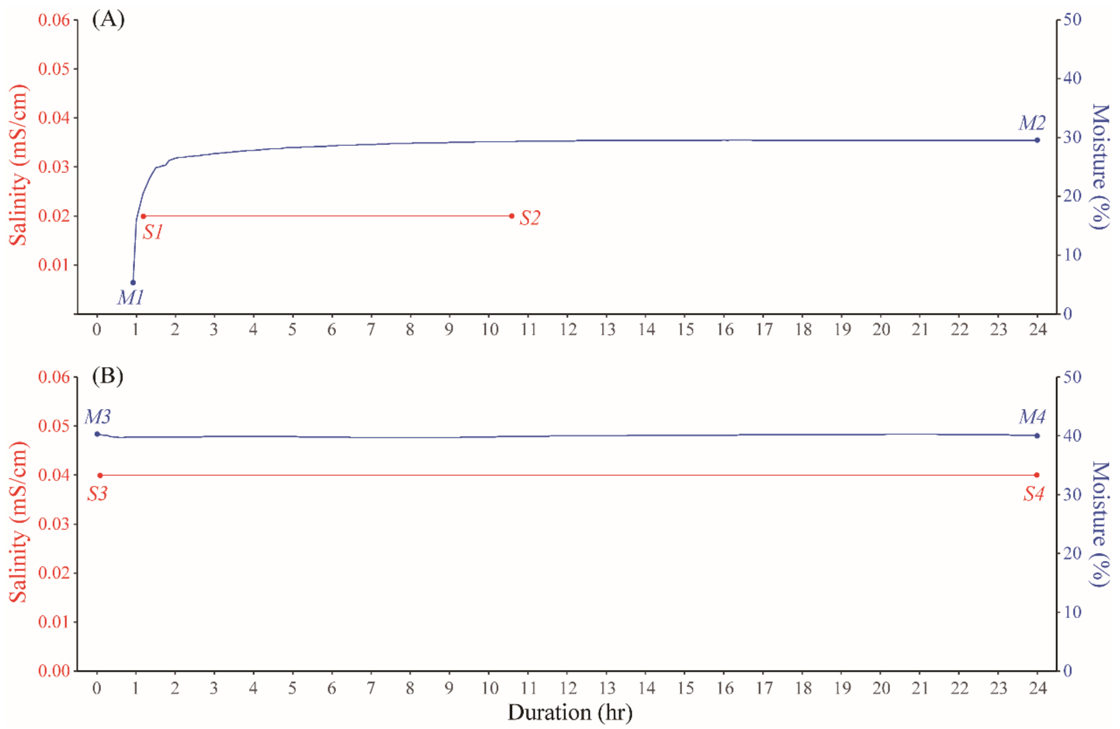

Figure S2. Results (non-zero values) of 24-hour monitoring for the diffusion of water and nutrient ions (indicated by salinity) between mesh-divided compartments. The water and salinity sensors were placed in the donor compartment, where results were recorded. After pouring the nutrient solution into the donor compartment, the first appearance of a non-zero salinity value indicated that nutrient ions from the solution had diffused passing through the mesh to the location where the sensor was placed. The first appearance of non-zero moisture value in the dry test indicated that water from the donor compartment had also diffused passing through the mesh to the local where the moisture sensor was place. In the dry test (A) the first non-zero value (5.3%) of soil moisture was recorded at the time point M1 (55 min) and that (0.02) of soil salinity was recorded at the time point S1 (70 min). The moisture value stabilized around 29% to 30% after 450 min; while interestingly the value of soil salinity returned to zero after the time point S2 (635 min). In the wet test (B), the first non-zero value (0.04) of soil salinity was recorded (by manual) at the time point S3 (3 min), and it lasted to the end of the test. For the whole duration of monitoring, the value of soil moisture was stabilized around 40%.

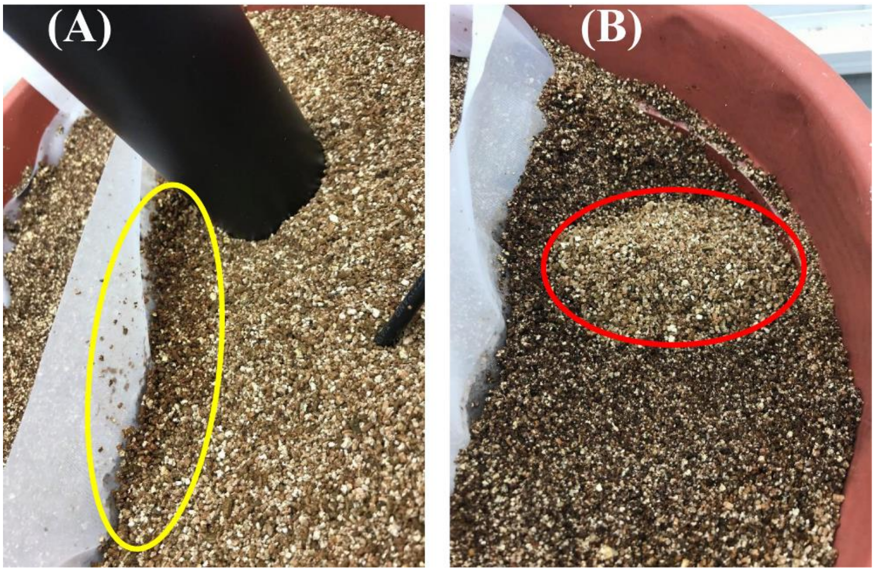

Figure S3. The diffusion of water within 24 hours in the dry test using a mesh-divider. (A) One hour after the beginning of test, we have already observed a clear sign of water diffusion passing through the mesh in the dry compartment (highlighted by a yellow circle). (B) In the end of the test (24 hours later), water had diffused all over the dry compartment. After the test, we added some dry vermiculites (highlighted by a red circle) for colour comparison (i.e. dry vs. moist vermiculite).

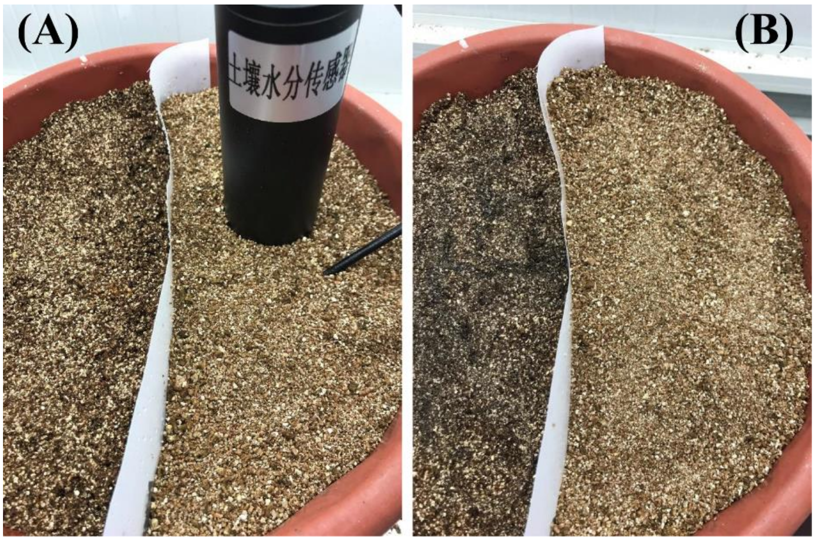

Figure S4. No sign of water diffusion in the dry test using a plastic film-divider. (A) One hour after the beginning of monitoring and (B) in the end of the monitoring (24 hours later), all vermiculite in the receiver compartment always remained dry. Contrast to the results of tests using mesh dividers (Figure S3), the colour of dry vermiculite in the receiver compartment (on the left side of the divider) remained unchanged and was clearly different from that of wet vermiculite in the donor compartment (on the right side).
